# Hospital infection control in hematopoietic stem cell transplant recipients.

**DOI:** 10.3201/eid0702.010223

**Published:** 2001

**Authors:** C A Dykewicz

**Affiliations:** Centers for Disease Control and Prevention, Atlanta, Georgia 30333, USA. cad3@cdc.gov

## Abstract

Guidelines for Preventing Opportunistic Infections Among Hematopoietic Stem Cell Transplant Recipients contains a section on hospital infection control including evidence-based recommendations regarding ventilation, construction, equipment, plants, play areas and toys, health-care workers, visitors, patient skin and oral care, catheter-related infections, drug-resistant organisms, and specific nosocomial infections. These guidelines are intended to reduce the number and severity of hospital infections in hematopoietic stem cell transplant recipients.


### Hospital Infection Control in Hematopoietic Stem Cell Transplant Recipients

**Clare A. Dykewicz**
Centers for Disease Control and Prevention, Atlanta, Georgia, USA

Guidelines for Preventing Opportunistic Infections Among Hematopoietic Stem Cell Transplant Recipients contains a section on hospital infection control including evidence-based recommendations regarding ventilation, construction, equipment, plants, play areas and toys, health-care workers, visitors, patient skin and oral care, catheter-related infections, drug-resistant organisms, and specific nosocomial infections. These guidelines are intended to reduce the number and severity of hospital infections in hematopoietic stem cell transplant recipients.

The Centers for Disease Control and Prevention (CDC), the Infectious Diseases Society of America (IDSA), and the American Society for Blood and Marrow Transplantation (ASBMT) sponsored the Guidelines for Preventing Opportunistic Infections Among Hematopoietic Stem Cell Transplant Recipients. This document was drafted in 1997 by a working group of infectious disease and transplant experts,[1] revised extensively from 1997 to 1999, and released for public comment on September 15, 1999, on the CDC website. The final document was published in CDC's Morbidity and Mortality Weekly Report on October 20, 2000, and in the Biology of Blood and Marrow Transplantation in late 2000. The term hematopoietic stem cell transplant recipients (HSCT) is preferable to "bone marrow transplant recipients" because the new term more accurately describes the current state of transplantation, which may involve harvesting donor cells from peripheral blood, umbilical cord blood, or bone marrow (1).

The document is an evidence-based statement of recommended strategies for preventing opportunistic infections in HSCT recipients. The prevention strategies are rated by the strength of the recommendation and the quality of the evidence supporting it. This rating system was developed by IDSA and the U.S. Public Health Service for use in the guidelines for the prevention of opportunistic infections in persons infected with HIV (2). The rating system allows the importance of each recommendation to be assessed. An A rating indicates strong evidence for efficacy and clinical benefit and an intervention that should always be offered; an intervention with a B rating is supported by moderate evidence and generally should be offered; a C rating indicates an optional intervention because evidence is insufficient to support a recommendation or evidence for efficacy might not outweigh adverse effects; a D rating indicates that moderate evidence for lack of efficacy or adverse outcome supports recommending against the intervention; and an E rating indicates strong evidence that an intervention is contraindicated because of lack of efficacy or adverse effects. Three categories are used to rate the quality of evidence supporting each recommendation, with I the highest, indicating evidence from at least one randomized, controlled trial; II indicating evidence from at least one well-designed clinical trial without randomization, from cohort or case-controlled analytic studies, or from multiple time-series, or dramatic results from uncontrolled experiments; and III indicating evidence from authorities' opinions based on clinical experience, descriptive studies, or reports of expert committees. This article summarizes the hospital infection control guidelines in the Guidelines for Preventing Opportunistic Infections Among Hematopoietic Stem Cell Transplant Recipients, with ratings in brackets.

Address for correspondence: Clare A. Dykewicz, Centers for Disease Control and Prevention, 1600 Clifton Rd., N.E., Mailstop A12, Atlanta, GA 30333, USA; fax: 404-639-4664; e-mail: cad3@cdc.gov

### Ventilation

All allogeneic HSCT recipients should be placed in rooms with >12 air exchanges per hour (3,4) and point-of-use, high-efficiency (>99%) particulate air (HEPA) filters capable of removing particles $\geq$0.3 µm in diameter (4-7) [AIII]. This recommendation is particularly important for facilities undergoing construction and renovation (8). The need for environmental HEPA filtration for autologous HSCT recipients has not been established; however, the use of HEPA-filtered rooms should be considered for autologous HSCT recipients who have prolonged neutropenia, the major risk factor for nosocomial aspergillosis [CIII].

The use of laminar air flow rooms for bone marrow transplant recipients has been controversial. Such rooms contain filtered air that moves in parallel, unidirectional flow; the air enters the room from one wall and exits the room on the opposite wall (3). Although LAF protects patients from infection in aspergillosis outbreaks during hospital construction (9,10), its routine use may not be valuable for all HSCT recipients (11). Since 1983, rooms with laminar air flow have been preferred for allogeneic HSCT recipients with aplastic anemia and human leukocyte antigen-identical sibling donors because the reported death rate of patients in regular rooms was nearly four times higher (12). However, the survival of aplastic anemia HSCT recipients in the late 1990s exceeds that reported in the early 1980s, and no study has yet

[1]Chair: Clare A. Dykewicz: Members: Raleigh A. Bowden, David Emanuel, David Longworth, Philip A. Rowlings, Robert H. Rubin, Kent A. Sepkowitz, Keith Sullivan, and John R. Wingard. CDC members: Robert T. Chen, Brian R. Edlin, Beth Hibbs, Harold W. Jaffe, William R. Jarvis, Jonathan Kaplan, Thomas J. Spira.

determined whether survival of HSCT recipients with aplastic anemia improves when they are treated in rooms with laminar air flow. Therefore, such rooms need not be constructed for every HSCT recipient, and use of available rooms is optional [CII].

Hospital rooms should have directed airflow so that air enters at one side of the room and is exhausted at the opposite side (5) [BIII]. Each hospital room should be well sealed (e.g., around windows and electrical outlets) (5) [BIII]. To provide consistent positive pressure in the HSCT recipient's room, consistent pressure differentials should be maintained between patients' rooms and the hallways or anterooms at >2.5 Pascals (3,4) [BIII]. In general, air pressure in hospital rooms of HSCT recipients should be higher than in adjoining hallways, toilets, and anterooms.

Backup emergency power and redundant systems should be provided to maintain room pressurization and a constant number of air exchanges in HSCT units when the central ventilation system is shut off for maintenance and repair (13) [BIII]. In addition, protocols should be developed to protect HSCT units from bursts of mold spores when air-handling systems are restarted after routine maintenance [BIII].

### Construction

Hospital construction and renovation have been associated with increased risk for nosocomial fungal infection, especially aspergillosis, among severely immunocompromised patients (14). Therefore, people responsible for HSCT unit construction or renovation should consult published recommendations for environmental controls (15,16) [AIII]. Planning for construction or renovation should include strategies for intensified aspergillosis-control measures [AIII]. The planning committee should include engineers, architects, housekeeping staff, infection control personnel, the director of the HSCT unit, administration representatives, and safety officers [BIII].

### Isolation

HSCT units should follow published guidelines for hospital isolation practices, including CDC guidelines for the prevention of nosocomial infections (17,18) [AIII]. However, the efficacy of specific isolation and barrier precautions in preventing nosocomial infections in HSCT recipients has not been evaluated. HSCT recipients should be placed in private rooms [BIII]. When indicated, HSCT recipients should also be placed on airborne, droplet, or contact precautions in addition to standard precautions (17) [AIII]. Careful observation of isolation precautions is important to prevent transmission of infectious agents among HSCT recipients, health-care workers, and visitors.

### Hand Hygiene

Hand hygiene is the single most effective procedure for preventing nosocomial infection (17). Everyone, especially health-care workers, should wash hands before entering and after leaving rooms of HSCT recipients and candidates undergoing conditioning therapy (chemotherapy and radiation) (17,19) or before and after any direct contact with patients, regardless of whether hands were soiled [AI]. HSCT recipients should be encouraged to practice good hand hygiene (e.g., washing hands before eating, after using the toilet, before and after touching a wound) [BIII]. Hands should be washed with antimicrobial soap and water [AIII]; hygienic hand rubs are also an acceptable means of maintaining hand hygiene (20,21). Health-care workers wearing gloves should put them on in the patient's room after handwashing and then discard them in the same patient's room before washing hands again on exiting the room. Gloves should always be changed between patients or before touching a clean area if the gloves become soiled (e.g., change gloves after touching the perineum and before touching a clean area) [AIII]. Appropriate gloves should be used by all persons handling potentially contaminated biological materials [AII].

### Equipment

HSCT units should monitor opened and unopened wound-dressing supplies such as adhesive bandages (22) and surgical and elastic adhesive tape (23) to detect mold contamination and prevent cutaneous transmission to patients [BII]. All bandages and wound dressings should be discarded that are out of date, have damaged packaging, or are visually contaminated by construction debris, moisture. [BIII].

### Plants

Exposure to plants and flowers has not been conclusively shown to cause fungal infections in HSCT recipients. However, most experts strongly recommend that plants and dried or fresh flowers not be allowed in the hospital rooms of HSCT recipients or candidates undergoing conditioning therapy because *Aspergillus* spp. have been isolated from the soil of potted ornamental plants (e.g., cacti), the surface of dried flower arrangements, and fresh flowers (5,7,24) [BIII].

### Play Areas and Toys

Play areas for pediatric HSCT recipients and candidates undergoing conditioning therapy should be cleaned and disinfected weekly and as needed [BIII]. Only toys, games, and videos that can be kept clean and disinfected should be allowed in the HSCT unit [BIII]. HSCT units and clinics should follow published recommendations for washing and disinfecting toys (25) [BIII].

### Health-Care Workers

Each hospital or HSCT center should prepare a written comprehensive policy on the immunization of hospital personnel that meets current recommendations of CDC, the Advisory Committee on Immunization Practices, and the Healthcare Infection Control Practices Advisory Committee (26) [BIII]. Immunizations are needed to prevent transmission of vaccine-preventable diseases to HSCT recipients and candidates undergoing conditioning therapy. In general, health-care workers should be immune to measles, mumps, rubella, and especially varicella and influenza.

### Visitors

Hospitals should have written policies for screening HSCT unit visitors, especially children, for potentially infectious conditions. Such screening should be performed by clinically trained health-care personnel [BII]. Visitors who have communicable infectious diseases such as upper respiratory infection or flulike illness, recent exposure to communicable diseases, an active shingles rash (whether covered or not), a *Varicella zoster*-like rash within 6 weeks of receiving a chickenpox vaccine, or a history of receiving an oral polio vaccine within the previous 3 to 6 weeks should not be allowed to enter the HSCT unit or have direct contact with HSCT recipients or candidates undergoing conditioning therapy [AII].

### Patient Skin Care

Skin care during neutropenia should include daily inspection of sites likely to be portals of infection, such as the perineum and intravascular access sites [BIII]. HSCT recipients and candidates undergoing conditioning therapy should maintain good perineal hygiene to minimize loss of skin integrity and risk for infection [BIII]. To facilitate this, HSCT units should develop special protocols for patient perineal care. To prevent vaginal or cervical irritation and abrasions, menstruating immunosuppressed HSCT recipients should not use tampons [DIII]. (Immunosuppressed HSCT recipients are defined as being <24 months post-HSCT, on immunosuppressive therapy, or having graft-versus-host disease.) The use of rectal thermometers, enemas, suppositories, and rectal exams are contraindicated for HSCT recipients because of the risk for skin or mucosal breakdown [DIII].

### Oral and Dental Care

Establishing optimal periodontal health before HSCT is one of the most important steps patients can take to avoid oral infections, and maintaining good oral hygiene after the transplant can minimize the severity and facilitate healing of mucositis, especially before engraftment [BIII]. All HSCT candidates should receive a dental evaluation and relevant treatment before conditioning therapy begins (27) [AIII]. Likely sources of dental infection should be rigorously eliminated [AIII].

HSCT recipients with mucositis and HSCT candidates undergoing conditioning therapy should maintain good oral hygiene by rinsing the mouth four to six times a day with sterile water, normal saline, or sodium bicarbonate solutions (27) [AIII]. HSCT recipients and candidates should brush their teeth at least twice a day with a soft regular toothbrush (27) [BIII]. Patients who cannot tolerate these brushings may use ultra-soft toothbrushes or sponge or foam toothettes (Sage Products, Crystal Lake, IL) [CIII], but these products are less effective in removing dental debris (17). Toothpaste is optional, depending on patient tolerance (27) [CIII]. HSCT recipients and candidates undergoing conditioning therapy who are skilled at dental flossing should floss daily if this can be done without trauma [BIII].

### Prevention of Bacterial Infections Related to Intravascular Catheters

HSCT units are advised to implement published guidelines for preventing infections related to the use of intravascular devices (28) [AIII]. HSCT units should avoid tap-water contact with the central venous catheter site [BIII]. To prevent bloodstream infections associated with the use of needleless intravenous-access devices, HSCT recipients should cover and protect the catheter tip or end cap during bathing or showering to protect it from tap-water contamination, change the device in accordance with manufacturers' recommendations, and have a care giver perform IV infusions whenever possible (29) [BII].

### Drug-Resistant Organisms

Avoiding the misuse of antibiotics will decrease the emergence of drug-resistant strains of bacteria. Therefore, HSCT units should routinely review patterns of use for antibiotics and should prudently prescribe all antibiotics, especially vancomycin, to prevent the emergence of multidrug-resistant organisms. Medical and ancillary staff members responsible for monitoring antimicrobial use patterns should routinely review vancomycin use (30) [AIII]. Vancomycin and all other antibiotics, especially third-generation cephalosporins and antianaerobic agents such as metronidazole, must be used judiciously (30) [AII].

### Specific Nosocomial Infections

Nosocomial pathogens are potential threats to all patients; however, if infected, HSCT recipients are at risk for more severe disease. Nosocomial pathogens of concern include *Legionella* spp., methicillin-resistant *Staphylococcus aureus*, *Streptococcus viridans*, and *Mycobacterium tuberculosis*, and community respiratory viruses such as influenza, respiratory syncytial virus, adenovirus, and parainfluenza virus.

#### Legionellosis

Clinicians should always consider infection with *Legionella* spp. in the differential diagnosis of pneumonia in HSCT recipients. Because HSCT recipients are at much higher risk for disease and death from legionellosis (31), periodic routine culturing for legionellae in water samples from the transplant units' potable water supply may be part of an overall prevention strategy in such units [CIII]. However, the optimal methods (frequency, number of sites) for environmental surveillance cultures in transplant units have not been determined, and the cost-effectiveness of this strategy has not been evaluated. Because HSCT recipients are at high risk for legionellosis and a safe concentration of legionellae organisms in potable water has not been determined, the goal, if environmental surveillance is undertaken, should be to maintain water systems with no detectable organisms [AIII]. Clinicians must maintain a high index of suspicion for legionellosis in transplant patients with nosocomial pneumonia even when environmental surveillance cultures do not yield legionellae [AIII].

### Community Respiratory Virus Infections

Clinicians should institute appropriate precautions and infection control measures to prevent nosocomial pneumonia in hospitalized HSCT recipients and candidates undergoing conditioning therapy, especially during community or nosocomial respiratory virus outbreaks (5) [AIII]. Even when there is no nosocomial or community outbreak of respiratory virus infections, which are emerging infections in HSCT recipients, everyone who enters an HSCT unit, including visitors and health-care workers, should be screened daily for symptoms of upper respiratory infection [BIII]. Some experts recommend that health-care workers who work in HSCT units should provide daily verification (e.g., sign-in sheets) that they are symptom free before being allowed to care for patients. To minimize the risk for transmission, health-care workers and visitors with upper respiratory symptoms should be restricted from contact with HSCT recipients and candidates undergoing conditioning therapy [AIII]. All health-care workers with upper respiratory infection symptoms should be restricted from patient contact and reassigned to nonpatient care duties until their symptoms resolve [BIII]. Visitors with such symptoms should be asked to defer their visit to the HSCT unit until their symptoms resolve [BIII].

Viral shedding among HSCT recipients with community respiratory virus infection has been documented to last up to 4 months for influenza (32), 2 years for adenovirus (33), and 22 days for respiratory syncytial virus (34); however, viral shedding has been reported to last up to 112 days in a child with severe combined immunodeficiency (35). Therefore, to prevent nosocomial transmission, HSCT units should factor such possible prolonged viral shedding into policy decisions about duration of precautions for infected HSCT recipients or candidates undergoing conditioning therapy [CIII].

### *Mycobacterium tuberculosis*

HSCT candidates should be screened for tuberculosis (TB) by a careful medical history and chart review to ascertain any history of TB exposure [AIII] because latent TB infection is more likely to progress to active disease among persons who are immunocompromised (36). HSCT units should also consider administering a tuberculin skin test (TST) by the Mantoux method with 5 tuberculin units of purified protein derivative (PPD) [CIII]; however, the TST may not be reliable in immunocompromised patients. Patients with a recent positive TST result or a history of a positive TST result and no prior preventive therapy should be given a chest X ray and evaluated for active TB (36) [AI]. Because immunocompromised patients have a decreased ability to mount a delayed hypersensitivity response, a positive TST result for them is defined as $\geq$5 mm of induration (36) rather than $\geq$10 mm [CIII]. Since immunosuppressive therapy decreases the sensitivity of the TST, HSCT providers should not rely solely on the TST to determine presence of latent TB infection and need for preventive therapy [DIII]. Instead, a full 9-month course of isoniazid preventive therapy should be given to immunocompromised HSCT recipients or candidates who have had close contact with someone with active, infectious (i.e., sputum-smear positive) pulmonary or laryngeal TB, regardless of the HSCT recipient's or candidate's TST status (36) [BIII]. Routine anergy screening results may not be reliable for HSCT recipients and candidates undergoing conditioning therapy, and therefore such screening is not recommended [DIII]. HSCT should not be canceled or delayed because of a positive TST result [DIII].

### Infection Control Surveillance

HSCT units should not perform routine fungal or bacterial cultures of asymptomatic HSCT recipients (37) [DII]. In the absence of epidemiologic clusters of infections, HSCT units should not perform routine periodic bacterial surveillance cultures of the HSCT unit environment or of equipment or devices used for respiratory therapy, pulmonary-function testing, or delivery of inhalation anesthesia (5) [DIII]. Some experts suggest that hospitals routinely sample air, ceiling tiles, ventilation ducts, and filters to test for molds, especially when construction or renovation occurs near or around the rooms of immunocompromised patients (24,37) or when clinical surveillance demonstrates a possible increase in mold (e.g., aspergillosis) cases [CIII]. In the absence of a nosocomial fungal outbreak, HSCT units need not perform routine fungal cultures of devices and dust in the rooms of HSCT recipients and candidates undergoing conditioning therapy [DIII]. HSCT units should routinely monitor the number of aspergillosis cases occurring in HSCT recipients, especially during hospital construction or renovation [BIII]. A twofold or greater increase in the attack rate of aspergillosis during any 6-month period indicates that the HSCT unit environment should be evaluated for breaks in infection control techniques and procedures and that the ventilation system should be carefully investigated (21) [BIII].

Careful adherence to the recommendations in these Guidelines for the Prevention of Opportunistic Infections in Hematopoietic Stem Cell Transplant Recipients may decrease the rate of hospital infections among HSCT recipients.

### Acknowledgments

The author thanks Richard Besser, John M. Boyce, Raymond Chinn, Harold W. Jaffe, William R. Jarvis, Jonathan E. Kaplan, William Kohn, David L. Longworth, Michael McNeil, Lauren Patton, Doug Peterson, Michele Pearson, Frank Rhame, Renee Ridzon, Kent A. Sepkowitz, Jane D. Siegel, Andrew J. Streifel, Raymond Strikas, Keith Sullivan, Thomas Walsh, and Estella Whimbey for their assistance.

Dr. Dykewicz is a medical epidemiologist in the Division of AIDS, STD, and TB Laboratory Research, National Center for Infectious Diseases, CDC. She was an officer in the Epidemic Intelligence Service from 1989 to 1991 and completed a fellowship in pediatric infectious diseases at Yale University in 1995.

### References

1. Dykewicz CA. Preventing opportunistic infections in bone marrow transplant recipients. Transplant Infectious Disease 1999;1:40-9.
2. United States Public Health Service (USPHS)/Infectious Diseases Society of America (IDSA) Prevention of Opportunistic Infections Working Group. USPHS/IDSA guidelines for the prevention of opportunistic infections in persons infected with human immunodeficiency virus: a summary. MMWR Morb Mortal Wkly Rep 1995;44(No.RR-8):1-34. Available from: URL: http://www.cdc.gov/epo/mmwr/preview/mmwrhtml/00038328.htm
3. Streifel AJ. Design and maintenance of hospital ventilation systems and the prevention of airborne nosocomial infections. In: Mayhall CG, editor. Hospital epidemiology and infection control. Philadelphia: Lippincott, Williams & Wilkins; 1999. p. 1211-21.
4. Streifel AJ, Marshall JW. Parameters for ventilation controlled environments in hospitals. In: Moschandreas DJ, editor. Design, construction and operation of healthy buildings. Solutions to global and regional concerns. Atlanta: American Society of Heating, Refrigeration, and Air-Conditioning Engineers Press; 1998. p. 305-9.
5. Centers for Disease Control and Prevention. Guidelines for prevention of nosocomial pneumonia. MMWR Morb Mortal Wkly Rep 1997;46(RR-1):1-79,or Respir Care Clin N Am 1994;39:1191-236, or available at URL: http://www.cdc.gov/epo/mmwr/preview/ind97_rr.html
6. American Institute of Architects Academy of Architecture for Health. 1996-1997 Guidelines for design and construction of hospitals and health care facilities. Washington: The Institute; 1996.
7. Rhame FS, Streifel AJ, Kersey JH Jr, McGlave PB. Extrinsic risk factors for pneumonia in the patient at high risk of infection. Am J Med 1984;76(Suppl 5A):42-52.
8. Opal SM, Asp AA, Cannady PB Jr, Morse PL, Burton LJ, Hammer PG II. Efficacy of infection control measures during a nosocomial outbreak of disseminated aspergillosis associated with hospital construction. J Infect Dis 1986;153:634-7.
9. Barnes RA, Rogers TR. Control of an outbreak of nosocomial aspergillosis by laminar air-flow isolation. J Hosp Infect 1989;14:89-94.

10. Sheretz FJ, Belani A, Kramer BS, Elfenbein GJ, Weiner RS, Sullivan ML, et al. Impact of air filtration on nosocomial aspergillus infections. Unique risk to bone marrow transplant recipients. Am J Med 1987;83:709-18.
11. Walter EA, Bowden RA. Infection in the bone marrow transplant recipient. Infect Dis Clin N Am 1995;9:823-47.
12. Storb R, Prentice RL, Buckner CD, Clift RA, Appelbaum F, Deeg J, et al. Graft-versus-host disease and survival in patients with aplastic anemia treated by marrow grafts from HLA-identical siblings. N Engl J Med 1983;308:302-7.
13. Streifel AJ. Maintenance and engineering. In: Association for Professionals in Infection Control and Epidemiology, Inc. Infection control and applied epidemiology: principles and practice. 2nd edition. St. Louis: Mosby; 2000. p. 76-1.
14. Weems JJ Jr, Davis BJ, Tablan OC, Kaufman L, Martone WJ. Construction activity: an independent risk factor for invasive aspergillosis and zygomycosis in patients with hematologic malignancy. Infect Control 1987;8:71-5.
15. Vesley D, Streifel AJ. Environmental services. In: Mayhall CB, editor. Hospital epidemiology and infection control. 2nd ed. Philadelphia: Lippincott, Williams, & Wilkins; 1999. p. 1047-53.
16. Carter CD, Barr BA. Infection control issues in construction and renovation. Infect Control Hosp Epidemiol 1997;18:587-96. Available at URL: http://www.cdc.gov/epo/mmwr/preview/mmwrhtml/00035909.htm
17. Garner JS, the Hospital Infection Control Practices Advisory Committee. Guideline for isolation precautions in hospitals. Infect Control Hosp Epidemiol 1996;17:1-80.
18. Centers for Disease Control and Prevention. Overview of CDC Guidelines for the Prevention and Control of Nosocomial Infections. Available at URL: http://www/cdc/gov/ncidod/hip/Guide/overview.htm
19. Garner JS, Favero MS. CDC Guidelines for the prevention and control of nosocomial infections. Guideline for handwashing and hospital environmental control, 1985. Supersedes guideline for hospital environmental control published in 1981. Am J Infect Control 1986;14:110-29, or available at URL: http://www.cdc.gov/ncidod/hip/Guide/handwash.htm
20. Rotter ML. Hand washing and hand disinfection. In: Mayhall CG, editor. Hospital epidemiology and infection control. 2nd ed. Baltimore: Lippincott, Williams, & Wilkins; 1999. p. 1339-55.
21. Larson EL. APIC Guideline for handwashing and hand antisepsis in health care settings. Am J Infect Control 1995;23:251-69.
22. Centers for Disease Control and Prevention. Nosocomial outbreak of *Rhizopus* infections associated with Elastoplast wound dressings—Minnesota. MMWR Morb Mortal Wkly Rep 1978;27:33-4.
23. Bryce EA, Walker M, Scharf S, Lim AT, Walsh A, Sharp N, et al. An outbreak of cutaneous aspergillosis in a tertiary-care hospital. Infect Control Hosp Epidemiol 1996;17:170-2.
24. Walsh TJ, Dixon DM. Nosocomial aspergillosis: environmental microbiology, hospital epidemiology, diagnosis and treatment. Eur J Epidemiol 1989;5:131-42.
25. Centers for Disease Control and Prevention. The ABCs of safe and healthy child care. Available at URL: http://www.cdc.gov/ncidod/hip/ABC/abc.htm
26. Centers for Disease Control and Prevention. Immunization of health care workers: recommendations of the Advisory Committee on Immunization Practices (ACIP) and the Hospital Infection Control Practices Advisory Committee. MMWR Morb Mortal Wkly Rep 1997;46:(RR-18):1-42, or available at URL: http://www.cdc.gov/nip/publications/ACIP-list.htm
27. Schubert MM, Peterson DE, Lloid ME. Oral complications. In: Thomas E, Blume KG, Forman SJ, editors. Hematopoietic cell transplantation. 2nd ed. Oxford: Blackwell Science, Inc.; 1999. p. 751-63.
28. Pearson ML. Hospital Infection Control Practices Advisory Committee. Guidelines for prevention of intravascular device related infections, July 1996. Am J Infect Control 1996;24:262-93, or available at URL: http://www.cdc.gov/ncidod/hip/iv/iv.htm
29. Do AN, Ray BJ, Bannerjee SN, Illian AF, Barnett BJ, Pham MH, et al. Bloodstream infection associated with needleless device use and the importance of infection-control practices in the home health care setting. J Infect Dis 1999;179:442-8.
30. Centers for Disease Control and Prevention. Recommendations for preventing the spread of vancomycin resistance: recommendations of the Hospital Infection Control Practices Advisory Committee (HICPAC). MMWR Morb Mortal Wkly Rep 1995;44(RR-12):1-13, or available at URL: <http://aepo-xdv-www.epo.cdc.gov/wonder/prevguid/m0039349/m0039349.htm>.
31. Kool JL, Fiore AE, Kioski CM, Brown EW, Benson RF, Pruckler JM, et al. More than 10 years of unrecognized nosocomial transmission of legionnaires' disease among transplant patients. Infect Control Hosp Epidemiol 1998;19:898-904.
32. Hayden FG. Prevention and treatment of influenza in immunocompromised patients. Am J Med 1997;102:55-60.
33. Hillis WO, Cooper MR, Bang FB. Adenovirus infection in West Bengal. I. Persistence of viruses in infants and young children. Indian J Med Res 1973;61:980-8.
34. Harrington RD, Hooton TM, Hackman RC, Storch GA, Osborne B, Gleaves CA, et al. An outbreak of respiratory syncytial virus in a bone marrow transplant center. J Infect Dis 1992;165:987-93.
35. Hall CB, Powell KR, MacDonald DE, Gala CL, Menegus ME, Suffin SC, et al. Respiratory syncytial virus infection in children with compromised immune function. N Engl J Med 1986;315:77-81.
36. American Thoracic Society and Centers for Disease Control and Prevention. Targeted tuberculin testing and treatment of latent tuberculosis infection. Am J Respir Crit Care Med 2000;161:S221-S247.
37. Walsh TJ. Role of surveillance cultures in prevention and treatment of fungal infections. National Cancer Institute Monogr 1990;9:43-5.
